# Multi-omics Analysis of a Fecal Microbiota Transplantation Trial Identifies Novel Aspects of Acute GVHD Pathogenesis

**DOI:** 10.1158/2767-9764.CRC-24-0138

**Published:** 2024-06-10

**Authors:** Armin Rashidi, Maryam Ebadi, Tauseef U. Rehman, Heba Elhusseini, David Kazadi, Hossam Halaweish, Mohammad H. Khan, Andrea Hoeschen, Qing Cao, Xianghua Luo, Amanda J. Kabage, Sharon Lopez, Sivapriya Ramamoorthy, Shernan G. Holtan, Daniel J. Weisdorf, Alexander Khoruts, Christopher Staley

**Affiliations:** 1 Clinical Research Division, Fred Hutchinson Cancer Center; and Division of Oncology, University of Washington, Seattle, Washington.; 2 Division of Hematology, Oncology, and Transplantation, Department of Medicine, University of Minnesota, Minneapolis, Minnesota.; 3 Department of Radiation Oncology, University of Washington and Fred Hutchinson Cancer Center, Seattle, Washington.; 4 Department of Medicine, University of Minnesota, Minneapolis, Minnesota.; 5 Department of Surgery, University of Minnesota, Minneapolis, Minnesota.; 6 Biostatistics Core, Masonic Cancer Center, University of Minnesota, Minneapolis, Minnesota.; 7 Division of Biostatistics and Health Data Science, School of Public Health, University of Minnesota, Minneapolis, Minnesota.; 8 Division of Gastroenterology, Hepatology, and Nutrition, Department of Medicine, University of Minnesota, Minneapolis, Minnesota.; 9 Metabolon Inc., Durham, North Carolina.; 10 Biotechnology Institute, University of Minnesota, St. Paul, Minnesota.; 11 Center for Immunology, University of Minnesota, Minneapolis, Minnesota.

## Abstract

Acute GVHD (aGVHD) is a major complication of allogeneic hematopoietic cell transplantation (alloHCT) associated with gut microbiota disruptions. However, whether therapeutic microbiota modulation prevents aGVHD is unknown. We conducted a randomized, placebo-controlled trial of third-party fecal microbiota transplantation (FMT) administered at the peak of microbiota injury in 100 patients with acute myeloid leukemia receiving induction chemotherapy and alloHCT recipients. Despite improvements in microbiome diversity, expansion of commensals, and shrinkage of potential pathogens, aGVHD occurred more frequently after FMT than placebo. Although this unexpected finding could be explained by clinical differences between the two arms, we asked whether a microbiota explanation might be also present. To this end, we performed multi-omics analysis of preintervention and postintervention gut microbiome and serum metabolome. We found that postintervention expansion of *Faecalibacterium*, a commensal genus with gut-protective and anti-inflammatory properties under homeostatic conditions, predicted a higher risk for aGVHD. *Faecalibacterium* expansion occurred predominantly after FMT and was due to engraftment of unique donor taxa, suggesting that donor *Faecalibacterium*-derived antigens might have stimulated allogeneic immune cells. *Faecalibacterium* and ursodeoxycholic acid (an anti-inflammatory secondary bile acid) were negatively correlated, offering an alternative mechanistic explanation. In conclusion, we demonstrate context dependence of microbiota effects where a normally beneficial bacteria may become detrimental in disease. While FMT is a broad, community-level intervention, it may need precision engineering in ecologically complex settings where multiple perturbations (e.g., antibiotics, intestinal damage, alloimmunity) are concurrently in effect.

**Significance:**

Post-FMT expansion of *Faecalibacterium*, associated with donor microbiota engraftment, predicted a higher risk for aGVHD in alloHCT recipients. Although *Faecalibacterium* is a commensal genus with gut-protective and anti-inflammatory properties under homeostatic conditions, our findings suggest that it may become pathogenic in the setting of FMT after alloHCT. Our results support a future trial with precision engineering of the FMT product used as GVHD prophylaxis after alloHCT.

## Introduction

The gut microbiota modulates both the efficacy and toxicity of cancer treatment, especially in the setting of immunotherapy (1). Fecal microbiota transplantation (FMT) from healthy donors has been used to promote response to immune checkpoint inhibitor therapy (2, 3) and abrogate treatment-associated colitis (4, 5). Allogeneic hematopoietic cell transplantation (alloHCT) is the most profound immunotherapeutic modality, replacing the patient's entire immune system with a healthy donor's immune system. A graft-versus-tumor effect is hoped to eradicate the residual cancer. However, a major complication of this procedure is acute GVHD (aGVHD), the clinical phenotype of an immune attack by the graft against host epithelial tissues, affecting nearly half of all patients and carrying an approximately 20% risk of mortality (6). Disruptions in the gut microbiota including loss of diversity (7, 8), loss of obligate anaerobic and butyrogenic commensal bacteria (9–12), and expansion of *Enterococcus* (13) and mucolytic bacteria (14) have been associated with increased risk for aGVHD or its mortality. FMT has been used with success for the treatment of refractory aGVHD (15). However, whether FMT can prevent aGVHD is unknown.

We recently reported the clinical endpoints of a randomized, double-blind, placebo-controlled phase II trial, where we administered oral, encapsulated, third-party FMT versus placebo at the time of neutrophil recovery (16). The intervention (FMT vs. placebo) was used in the prophylactic setting. Two independent cohorts were enrolled: (i) adults undergoing alloHCT (74 patients) and (ii) patients with acute myeloid leukemia (AML) undergoing induction chemotherapy (26 patients). Preplanned clinical endpoints of the trial included feasibility, safety, infections (primary endpoint; both cohorts), and aGVHD (secondary endpoint; alloHCT cohort). The rationale for choosing these endpoints was the established link between the gut microbiota and both local intestinal and systemic immunity in health and various disease settings (17). We demonstrated that FMT was safe, feasible, and ameliorated gut dysbiosis, but did not significantly reduce infections. The incidence of grade II–IV aGVHD was numerically higher after FMT, an unexpected result partly explained by the imbalance between GVHD prophylactic regimens between the two arms (16). Donor microbiota engraftment rate was approximately 30%, and in *post hoc* analysis, greater engraftment rates correlated with lower rates of grade II–IV aGVHD (18).

Other than relatively poorly characterized modulation of the gut microbiota, the mechanism of action of FMT is unclear. While microbial metabolites, including those derived from dietary elements, have their greatest impact locally in the gut, some of these metabolites are absorbed across the intestinal wall and can reach distant sites, with potential effect. Whether FMT-mediated microbiota modulation leads to systemic metabolomic changes is unknown. Longitudinal pretreatment and posttreatment stool and blood samples collected in our trial and the presence of a randomized placebo arm uniquely positioned us to address this knowledge gap. Inspired by the established role of the gut microbiota in regulating blood metabolites (19–21) and systemic immunity (22, 23), we hypothesized that FMT modulation of the gut microbiota may alter the blood metabolome, potentially mediating some of its clinical effects. To test this hypothesis, we profiled the gut microbiome and serum metabolome before and after treatment and performed multi-omics analysis.

## Materials and Methods

### Trial Design Summary (Reported Previously)

The trial protocol (ClinicalTrials.gov identifier: NCT03678493) was approved by the University of Minnesota Institutional Review Board and complied with local regulations and the Declaration of Helsinki. The protocol, design, eligibility criteria, procedures, antibiotic exposures, and aGVHD details were published previously (16). Briefly, adults undergoing inpatient, T-replete alloHCT for any indication (HCT cohort) or inpatient chemotherapy for AML (AML cohort) underwent nonstratified double-blinded randomization in a 2:1 ratio to receive third-party FMT or placebo in the form of five oral capsules taken at once after neutrophil recovery and at least 2 days after discontinuation of antibacterial antibiotics. Importantly, patients who developed grade II–IV aGVHD before they were ready to start study treatment did not receive treatment and were thus not included. Each patient received material manufactured using Good Manufacturing Practice protocols from one of the 4 donors (24). Each FMT capsule contained ≥1 × 10^11^ bacteria with ≥40% viability. We enrolled 74 patients with HCT (FMT, 49; placebo, 25) and 26 patients with AML (FMT, 18; placebo, 8). Stool samples were collected in 95% ethanol (25, 26) at baseline [before starting conditioning (HCT cohort) or chemotherapy (AML cohort)], pre-FMT/placebo, 10 days post-FMT/placebo, 28 days post-FMT/placebo, and at 9 months. Serum samples were obtained at baseline, pre-FMT/placebo, 28 days post-FMT/placebo, and at 9 months. Samples were not collected after the onset of grade II–IV aGVHD. Therefore, findings from the analysis of samples in this work are not caused by GVHD or its treatment.

### Gut Microbiome and Serum Metabolome Profiling

DNA was extracted using the DNeasy PowerSoil DNA isolation kit (QIAGEN). qPCR was used to quantify 16S rRNA gene content in each sample. Samples with >1,000 16S rRNA gene copies per µL (considered adequate in our pipeline) were sequenced. The V4 hypervariable region of the 16S rRNA gene was amplified on an Illumina MiSeq platform (2 × 300 paired-end mode; ref. 27). Exact amplicon sequence variants (ASV) were inferred using DADA2 v1.18.0 (28). For filtering, we used DADA2 default parameters (PHRED score threshold of 2, maximum number of expected errors of 2 for both forward and reverse reads) and truncation lengths of 220 (forward) and 200 (reverse). Dereplication, denoising, merging, and chimera removal were done using DADA2 default parameters. Taxonomic assignment was done by the naive Bayesian classifier implemented in DADA2 and the SILVA nonredundant v138.1 training set (29).

Serum samples were sent to Metabolon for untargeted, ultrahigh performance LC/MS-MS. Methodologic details followed Metabolon pipelines and were published previously (30). Compounds were identified by comparison to library entries of purified standards or recurrent unknown entities (31, 32). Metabolites were assigned to pathways based on three publicly available key chemical information resources: PubChem, Human Metabolome Database (HMDB), and Kyoto Encyclopedia of Genes and Genomes pathway database.

### Statistical Methods

All analyses were done on the HCT cohort only. The clinical phenotype of interest was grade II–IV aGVHD, defined per standard criteria (33). For patients with both 10- and 28-day post-FMT/placebo stool samples, only the 28-day sample was used for this analysis. The chosen sample is thereafter referred to as “early post-FMT/placebo,” to distinguish it from the 9-month sample. This choice followed our previous observation that FMT effects are not complete at 10 days and continue to mature in the following weeks (16). There were no differences between the two arms in the time of study treatment (FMT vs. placebo) initiation, and hence its temporally linked post-FMT/placebo samples (*P* = 0.18, Wilcoxon test). Stool samples with >1,000 sequence reads and ASVs with a relative abundance of >0.1% in at least two samples were selected. These ASVs were then collapsed at the genus level, and genera present in at least 1% of the samples were retained for analysis. In between-sample or beta diversity analysis, we used Aitchison distance (34) to measure compositional distance between samples. Group comparisons were made using permutational multivariate ANOVA (adonis test with 999 permutations; ref. 35) and ordination was visualized by principal coordinates analysis. Presence/absence-based similarity between ASV-level *Faecalibacterium* composition of samples was measured by binary similarity.

### Batch Normalization

Experimental protocols and laboratories remained unchanged throughout the trial. Because of the large number of samples, assays had to be done in more than one batch. Batch effect was minimized because of the study design. At the time of each batch analysis, all available samples were analyzed. As enrollment in the two arms and in the two cohorts occurred in parallel, no systematic bias was present in cohort/arm distribution among batches. The only exception was the last batch of stool samples which included mostly late post-FMT/placebo samples from the last several patients in the trial. As the focus of this study was on samples before aGVHD onset, the last batch did not contribute to our main findings. Serum samples were analyzed in two batches. One batch was considered the reference set and the other one the target set. Eighteen samples (“anchor samples”) were run in both batches, yielding values *x*_i_ (*i* = 1 to 18) in the reference batch and *y*_i_ (*i* = 1 to 18) in the target batch. The median of the ratios *x*_i_/*y*_i_ (*i* = 1 to 18) was calculated and used to batch-adjust the measurements in the target set.

### Topic Modeling and Lasso Regression

We considered two general scenarios for how gut microbiome alterations in relation with the treatment arm and grade II–IV aGVHD may be characterized: (i) by individual taxa, (ii) by microbial clusters. In scenario (i), FMT may increase/decrease the abundance of specific taxa, some of which might alter the risk of grade II–IV aGVHD. In scenario (ii), microbial subcommunities or clusters rather than individual taxa are the relevant microbial units. This concept is best exemplified by “enterotypes” (36), where each sample is described by one enterotype, or the more recent “enterosignatures” (37), where mixed membership is allowed and each sample is described by its fractional membership in different signatures. To cluster microbial signatures and samples simultaneously using unsupervised classification, we applied topic modeling (38) in topicmodels v.0.2-16. This approach, inspired from natural language processing and based on probabilistic latent variable models, considers samples as documents, microbial clusters as topics making the documents, and individual taxa as words making the topics. Latent Dirichlet allocation (LDA; ref. 39), one of the available algorithms, finds microbial taxa associated with each cluster, while also determining the mixture of clusters that describes each sample. The optimal number of clusters was chosen using a search between 2 and 10 topics per the Cao and colleagues method (40). An LDA model was then fitted using the Variational Exception Maximization (VEM) algorithm. Early post-FMT/placebo stool samples were used in this analysis.

To find taxon-level predictors (a.k.a features) of grade II–IV aGVHD in early post-FMT/placebo samples, we considered that the number of features far exceeded the number of events. To account for this property and the compositional nature of microbiome data, we used log-ratio lasso regression modeling and compositional feature selection as implemented in *FLORAL* v.0.2.0 (41). *FLORAL* uses an augmented Lagrangian algorithm for a zero-sum constraint optimization problem while enabling stepwise feature selection. Regression coefficients are estimated such that they meet the zero-sum constraint implemented by the algorithm. Four-fold cross-validation was used to select the penalty parameter (λ_min_). Considering the sample size and expected heterogeneity due to 4-fold data splits, 100 runs of cross validation were performed to rank the strength of the features by their selection probabilities. Selection of features in this method is based on their direct contribution to better prediction performances rather than arbitrary *P* value thresholds. Grade II–IV aGVHD was modeled as a binary outcome using a binomial distribution.

To find metabolomic predictors of grade II–IV aGVHD in early post-FMT/placebo samples, we used two lasso-based approaches. Lasso forces most coefficients to zero, leaving in only variables that are most likely true correlates of the outcome. First, grade II–IV aGVHD was modeled as a binary variable using a binomial distribution in 10-fold cross-validated logistic lasso regression, as implemented in *glmnet* v.4.1.7 (42). Lasso features were logarithmically transformed levels of noncollinear serum metabolites in early post-FMT/placebo samples. The GVHD prophylaxis regimen [posttransplant cyclophosphamide (PTCy)-based vs. other] was included as a covariate because it was not balanced between the two arms. Regression coefficients were chosen at the cross-validated minimum value of lambda (tuning factor) and averaged across validation folds. We performed stability analysis to ensure consistent direction of associations and the presence of the same significant features across different partitions of the dataset. To this end, the entire process described above was repeated 100 times, each time using a different random partitioning of the dataset for cross-validation. The probability across runs of being selected as a feature in the final model was calculated for each feature. As an alternative method, we used sparse partial least squares discriminant analysis (sPLS-DA; package *mixOmics*; ref. 43). Parameter tuning to find the optimal number of latent components to achieve the best classification performance based on the balanced error rate was done by 100 runs of 4-fold cross validation.

### Overall Analytic Perspective

Identifying independent relationships between variables in the patient populations studied in this work is very challenging. This is because of the multitude of factors that concurrently or sequentially influence the microbiota and clinical outcomes and may even interact with each other. Diet, antibiotics, chemoradiotherapy, immunosuppression, growth of an alloimmune system, GVHD prophylactic agents, and other medications are examples of such variables. Therefore, a high noise-to-signal ratio would inherently be present, increasing the risk of false discovery. To minimize this risk, our analyses were based on two principles: (i) We considered hypothesis generation rather than definitive conclusions as the main objective. Therefore, significant *P* values were considered evidence supporting further exploration rather than a proof, (ii) In multi-omics network analysis, we acknowledge the possible presence of intermediate mediators from microbiome to metabolome. Given the constraints mentioned above, we did not attempt to identify these mediators; rather, we sufficed to strong univariate correlations between the two compartments. The assumption here was that the strongest of such correlations may include mechanistic links and propose promising targets for future research.

### Data Availability Statement

The sequencing data reported in this article are available from NCBI Sequence Read Archive (SRA) under BioProject ID SRP287850. Any additional information required to reanalyze the data reported in this article is available from the corresponding author (Armin Rashidi, arashidi@fredhutch.org) upon request.

### Ethics Approval

The trial protocol (ClinicalTrials.gov identifier: NCT03678493) was approved by the University of Minnesota Institutional Review Board and complied with local regulations and the Declaration of Helsinki. All participants provided written informed consent.

## Results

**FIGURE 1:**
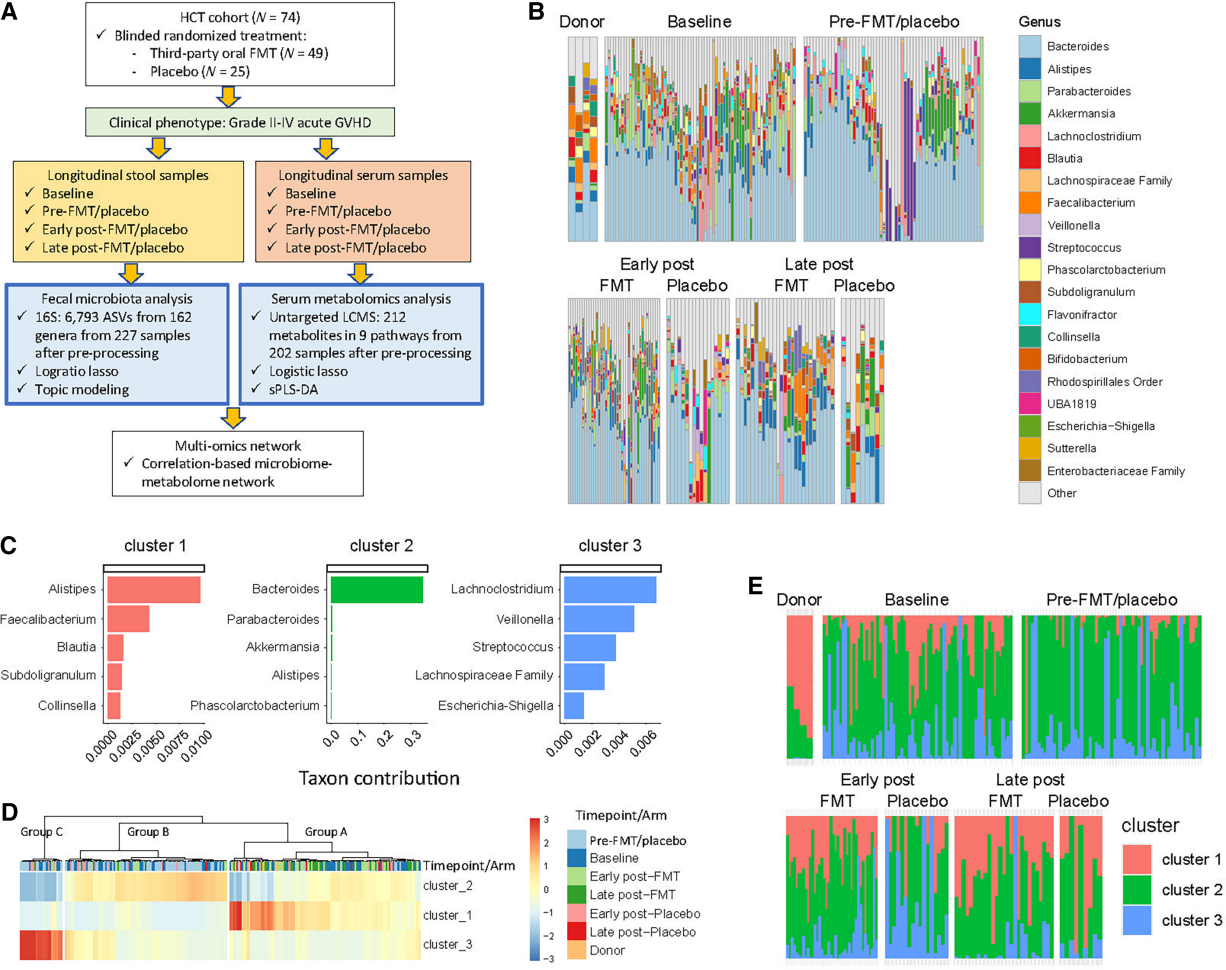
Microbial clusters in relation to treatment arm and timepoint. **A,** Overall study design and patient/sample breakdown. **B,** Genus-level taxonomic distribution among sample groups. Stacked bar charts show relative abundances of the most abundant genera among all stool samples. **C,** Microbial clusters and their top five contributing taxa from topic modeling. **D,** Cluster-level gut microbiota heatmap visualizing the results of topic modeling. A *ward.D* function was used to generate the heat map in *pheatmap* v.1.0.12. Each column is a sample, and each row is a cluster. The blue-red gradient shows cluster abundances scaled row-wise. Sample groups are defined by column dendrograms shown at the top. **E,** Cluster-level taxonomic distribution among sample groups. Stacked bar charts show relative abundances of the three clusters among all stool samples. In B and C, when genus-level taxonomy could not be reliably made, the best next level is shown. For example, “Lachnospiraceae Family” represents a genus in this family which could not be unambiguously assigned. ASV: amplicon sequence variant; FMT: fecal microbiota transplantation; GVHD: graft-versus-host disease; HCT: hematopoietic cell transplantation; LCMS: liquid chromatography-mass spectrometry; sPLS-DA: sparse partial least squares differential abundance analysis.

Baseline characteristics of the patients whose samples were analyzed in this work are provided in Table 1. Figure 1A provides an overview of multi-omics analysis and data. High-throughput profiling of the gut microbiota was performed using 16S rRNA gene short amplicon sequencing of the stool samples. Preprocessing (see above) filtered seven samples, yielding a total of 227 samples from 74 patients and containing a total of 6,793 ASVs mapped to 162 genera. The distribution of stool samples over time was donor (*N* = 4), baseline (*N* = 71), pre-FMT/placebo (*N* = 59), early postintervention [*N* = 40 (FMT); *N* = 17 (placebo)], and late postintervention [*N* = 27 (FMT); *N* = 9 (FMT)]. These numbers also reflect the numbers of patients analyzed at the corresponding timepoints as a maximum of one sample was available from each patient at each timepoint. Relative abundances of the 20 most abundant genera for the two treatment arms at each timepoint are shown in Fig. 1B.

**TABLE 1:** Baseline patient characteristics

Variable	FMT (*N* = 49)	Placebo (*N* = 25)	Total (*N* = 74)
Age at transplant – years			
Median (range)	57 (21–73)	52 (24–72)	55 (21–73)
Male sex – no. (%)	26 (53.1)	16 (64.0)	42 (56.8)
Underlying disease – no. (%)			
Acute leukemia	33 (67.3)	20 (80.0)	53 (71.6)
MDS/MPN	7 (14.3)	2 (8.0)	9 (12.2)
Nonmalignant disorders	6 (12.2)	2 (8.0)	8 (10.8)
CLL/NHL	3 (6.1)	1 (4.0)	4 (5.4)
HCT-CI – no. (%)			
Median (range)	2 (0–8)	2 (0–5)	2 (0–8)
0–1	22 (44.9)	8 (32.0)	30 (40.5)
2 or greater	27 (55.1)	17 (68.0)	44 (59.5)
HCT donor – no. (%)			
Matched unrelated	32 (65.3)	17 (68.0)	49 (66.2)
Matched sibling	14 (28.6)	6 (24.0)	20 (27.0)
Haploidentical	2 (4.1)	2 (8.0)	4 (5.4)
Cord blood	1 (2.0)	0 (0)	1 (1.4)
Conditioning intensity – no. (%)			
Reduced intensity	31 (63.3)	9 (36.0)	40 (54.1)
Myeloablative	18 (36.7)	16 (64.0)	34 (45.9)
HCT graft source – no. (%)			
Peripheral blood	40 (81.6)	21 (84.0)	61 (82.4)
Bone marrow	8 (16.3)	4 (16.0)	12 (16.2)
Cord blood	1 (2.0)	0 (0)	1 (1.4)
GVHD prophylaxis – no. (%)			
Tac/MMF/PTCy	22 (44.9)	19 (76.0)	41 (55.4)
MMF/Tac	25 (51.0)	6 (24.0)	31 (41.9)
Other	2 (4.1)	0 (0)	2 (2.7)
ATG – no. (%)			
Not used	36 (73.5)	21 (84.0)	57 (77.0)
Used	13 (26.5)	4 (16.0)	17 (23.0)
Dose 1, days from HCT			
Median (range)	23 (12–62)	26 (11–63)	24 (11–63)

Abbreviations: ATG, anti-thymocyte globulin; CI, comorbidity index; CLL, chronic lymphocytic leukemia; HCT, hematopoietic cell transplantation; FMT, fecal microbiota transplantation; MDS, myelodysplastic syndrome; MMF, mycophenolate mofetil; MPN, myeloproliferative neoplasm; NHL, non–Hodgkin lymphoma; PTCy, posttransplantation cyclophosphamide; Tac, tacrolimus.

### Microbial Signatures Associated with Treatment Arm and aGVHD

Topic modeling identified three microbial clusters (Fig. 1C). Main members of cluster 1 were *Alistipes* and *Faecalibacterium*, with smaller contributions from *Blautia*, *Subdoligranulum*, and *Collinsella*. Cluster 2 was characterized by *Bacteroides*. Cluster 3 included typical oral bacteria *Streptococcus* and *Veillonella*, as well as enteric pathogens of the Proteobacteria phylum such as *Escherichia-Shigella*. Other members of cluster 3 were *Lachnoclostridium* and Lachnospiraceae.

Hierarchical clustering using these clusters classified the samples into three groups, each characterized by enrichment in one cluster (Fig. 1D). Group A was enriched in cluster 1, group B in cluster 2, and group C in cluster 3. Visual qualitative examination of the cluster-based heat map suggested several patterns. First, group A was the most physiologic group. It included all 4 healthy FMT donors and only a few preintervention samples. The latter samples were collected at or shortly after the peak of antibiotic injury, and their rarity in group A was consistent with their nonphysiologic state. The dominance of cluster 1 in group A indicated enrichment of group A samples in several obligate anaerobic commensal bacteria representing cluster 1. Most post-FMT samples also belonged to this group, consistent with the restoring effect of FMT, shifting the microbiome toward states resembling healthy donors. Most of the late post-placebo samples but relatively few early post-placebo samples were in this group, consistent with partial spontaneous recovery of the microbiome in the long term. Groups B and C were enriched in pre-FMT/placebo and early post-placebo samples, consistent with dysbiosis and absence of a placebo effect. Considering the members of cluster 3, markers of dysbiosis in group C included ectopic colonization of oral bacteria and expansion of Proteobacteria. Baseline samples were scattered among groups A, B, and C, without an obvious predilection, suggesting varying levels of microbiome recovery from prior injuries in patients referred for alloHCT. Figure 1E shows the relative contribution of each cluster to samples in different groups. Clear patterns included: (i) dominance of cluster 1 and absence of cluster 3 among donors, (ii) shrinkage of cluster 1 from baseline to predose, (iii) partial correction of pattern (ii) after FMT, but not placebo. Specifically, FMT led to restoration of cluster 1 and shrinkage of cluster 3, and (iv) partial recovery of the microbiome after placebo, but only in the long-term.

**FIGURE 2:**
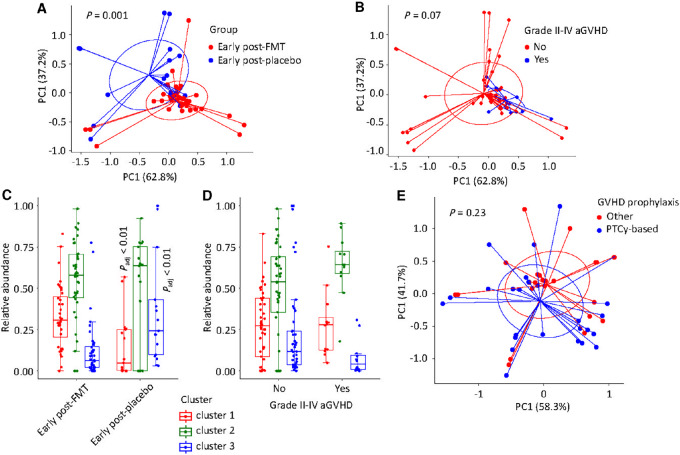
Microbiota clusters and aGVHD risk. **A,** Principal coordinates analysis of early post-FMT/placebo gut microbiota using topic model-based cluster abundances, with groups defined according to treatment arm. **B,** Same analysis as in A, but with groups defined according to subsequent development of grade II–IV aGVHD or not. **C,** Comparison between early post-FMT versus early post-placebo samples in topic model-based cluster abundances. **D,** Same analysis as in C, but with groups defined based on the occurrence of subsequent grade II–IV aGVHD. Samples are early post-FMT/placebo. **E,** Principal coordinates analysis of pre-FMT/placebo gut microbiota using topic model-based cluster abundances, with groups defined according to GVHD prophylaxis. *P* values in A, B, and E are from an adonis test with 999 permutations, with percent variation explained by each axis is shown in parentheses. 95% ellipses are shown. *P* values in C are from a Wilcoxon test after Bonferroni correction. Comparisons in D were not statistically significant. In C and D, each box shows the median (horizontal middle line) and interquartile range. Whisker lines indicate nonoutlier maximum and minimum values. A small jitter is included for better visualization. aGVHD: acute graft-versus-host disease; FMT: fecal microbiota transplantation; PC: principal coordinate; PTCy: posttransplantation cyclophosphamide.

FMT mediation of microbial signatures was further evaluated in principal coordinate analysis of early post-FMT/placebo samples using Aitchison distances derived from centered log-ratio (clr) abundances of the three microbial clusters. clr transformation considers the abundance of each element (here cluster) in the context of the abundance of every other element in the community. One advantage of clr-transformed values is that they are scale invariant, meaning the same ratio is expected to be obtained in a sample with few read counts or an identical sample with many read counts (44). This analysis showed a significant difference between early post-FMT versus early post-placebo samples (*P* = 0.001, adonis test with 999 permutations; Fig. 2A). A similar analysis comparing early post-FMT/placebo samples preceding grade II–IV versus no/grade I aGVHD suggested a microbial signature associated with aGVHD (*P* = 0.07; Fig. 2B). These findings raised the question of whether the signatures associated with FMT and grade II–IV aGVHD were the same. Demonstrating this would propose a microbiota-mediated mechanism for increased risk of grade II–IV aGVHD after FMT. We tested this hypothesis by comparing the percent contribution of each cluster with early postintervention samples in the two groups (FMT vs. placebo; grade II–IV vs. no/grade I aGVHD). The first comparison showed a higher abundance of cluster 1 and lower abundance of cluster 3 after FMT compared with placebo (Bonferroni-corrected *P* < 0.001 in both comparisons, Wilcoxon test; Fig. 2C). The second comparison did not find a significant difference between groups with versus without grade II–IV aGVHD (Fig. 2D). We also evaluated whether samples exposed to different GVHD prophylactic regimens showed different microbiota signatures. Such signatures would be most readily identified in pre-FMT/placebo samples. Focusing on these samples, principal coordinate analysis revealed no significant difference between samples from patients receiving PTCy-based GVHD prophylaxis versus others (Fig. 2E). Because of its multicollinearity with GVHD prophylaxis in our trial, conditioning intensity was not separately analyzed (16).

Overall, the results so far indicate that the gut microbiota signature of FMT is characterized by an expansion of cluster 1 and shrinkage of cluster 3. Taxonomic composition of these clusters indicated FMT's efficacy in restoring obligate anaerobic commensals and decolonization of the gut from enteropathogens and bacteria of presumed oral origin. However, these effects did not predict grade II–IV aGVHD risk.

### *Faecalibacterium* Expansion in the Gut, Occurring Predominantly After FMT, Predicts a Higher Risk for aGVHD

**FIGURE 3:**
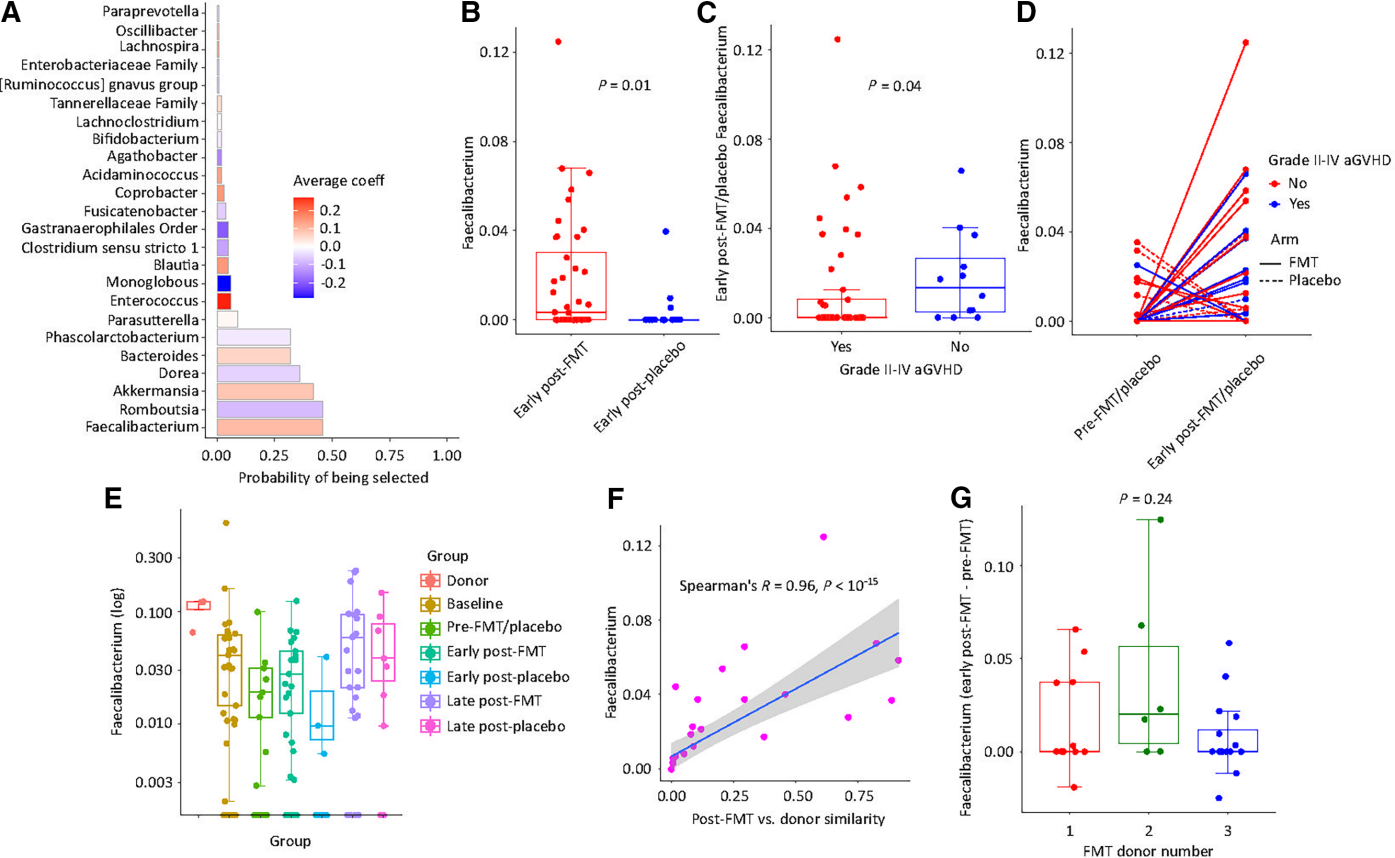
*Faecalibacterium* and aGVHD risk. **A,** log-ratio lasso regression to find early post-FMT/placebo, genus-level, gut microbiota predictors of grade II–IV aGVHD. The *y* axis shows taxa in the final model. The probability for each taxon of being selected in the final model is shown along the *x*-axis. This probability is calculated from 100 4-fold cross-validated runs. Positive/negative signs show positive/negative associations, and the value (indicated by a color gradient) represents the strength of association. **B,** Comparison between early post-FMT versus early post-placebo samples in *Faecalibacterium* abundance. **C,** Comparison between the groups developing versus not developing grade II–IV aGVHD in early post-FMT/placebo *Faecalibacterium* abundance. **D,***Faecalibacterium* abundance at pre-FMT/placebo and early post-FMT/placebo timepoints. Samples from the same patient are connected, with line type indicating randomized treatment arm and line color showing future grade II–IV aGVHD status. **E,***Faecalibacterium* abundance in FMT donor samples and patient samples according to treatment arm and timepoint. **F,** Relationship between *Faecalibacterium* abundance change (pre- to post-FMT) and ASV-level binary similarity of early post-FMT versus donor composition of this genus. The gray shaded area shows the 95% confidence interval. Higher post-FMT *Faecalibacterium* abundance was strongly associated with a greater similarity between post-FMT sample and donor composition in *Faecalibacterium* ASVs (a simple measure of engraftment). **G,***Faecalibacterium* change from pre-FMT to early post-FMT timepoints per FMT donor. Each box in B, C, and G shows the median (horizontal middle line) and interquartile range. Whisker lines indicate nonoutlier maximum and minimum values. A small jitter is included for better visualization. *P* values in B and C are from a Wilcoxon test and in G from a Kruskal–Wallis test. aGVHD: acute graft-versus-host disease; FMT: fecal microbiota transplantation.

Next, we asked whether a taxon-specific effect of FMT may lead to a higher risk of grade II–IV aGVHD. We performed cross-validated log-ratio lasso regression to identify the few genera that were most likely mediators of subsequent grade II–IV aGVHD risk (Fig. 3A). *Faecalibacterium* was the genus most strongly and consistently associated with grade II–IV aGVHD. *Faecalibacterium* was the second most contributory genus in cluster 1, the cluster that markedly expanded after FMT. This genus had a significantly higher relative abundance early after FMT than placebo (*P* = 0.01, Wilcoxon test; Fig. 3B) and in early post-FMT/placebo samples preceding grade II–IV versus no/grade I aGVHD (*P* = 0.04, Wilcoxon test; Fig. 3C). Within-subject changes in *Faecalibacterium* abundance before/after the intervention are visualized in Fig. 3D. There was a greater increase in *Faecalibacterium* abundance from preintervention to early postintervention in the FMT arm than the placebo arm (*P* = 0.06, paired Wilcoxon test) and in patients who subsequently developed grade II–IV versus no/grade I aGVHD (*P* = 0.04). As shown in Fig. 3E, *Faecalibacterium* was abundant in donor samples. Most patients had already lost much of their *Faecalibacterium* before conditioning initiation. This was aggravated during the initial phase of alloHCT, leading to further loss of *Faecalibacterium* until FMT/placebo. This decline pattern continued further after placebo but was largely reversed after FMT. *Faecalibacterium* recovery continued after FMT until the 9-month timepoint. Less robust long-term recovery occurred after placebo.

In our previous report, we showed that *Faecalibacterium* was the genus with the second highest engraftment rate from donor microbiota (16). These findings suggest that the high engraftment rate of *Faecalibacterium* combined with its enrichment in donor samples compared with pre-FMT patient microbiota contributed to its expansion after FMT, increasing the risk of aGVHD. To further evaluate this hypothesis, we calculated the binary similarity between early post-FMT microbiota and the corresponding donor microbiota. This index uses presence/absence of taxa and we applied it to ASVs which would represent taxonomy levels deeper than genus. We restricted our ASV table to variants mapped to *Faecalibacterium*. A greater binary similarity between two samples in this analysis would indicate a larger fraction of shared ASVs. We used this index as a simple measure of *Faecalibacterium* engraftment. We found a strong and positive correlation between post-FMT *Faecalibacterium* abundance and similarity of post-FMT *Faecalibacterium* community to the donor's (Spearman rho 0.96, *P* < 10^−15^; Fig. 3F). This finding demonstrates that post-FMT expansion of *Faecalibacterium* was due to *Faecalibacterium* engraftment. The extent of *Faecalibacterium* expansion after FMT did not depend on the donor used (Fig. 3G).

### FMT Does not Modulate the Blood Metabolome

**FIGURE 4:**
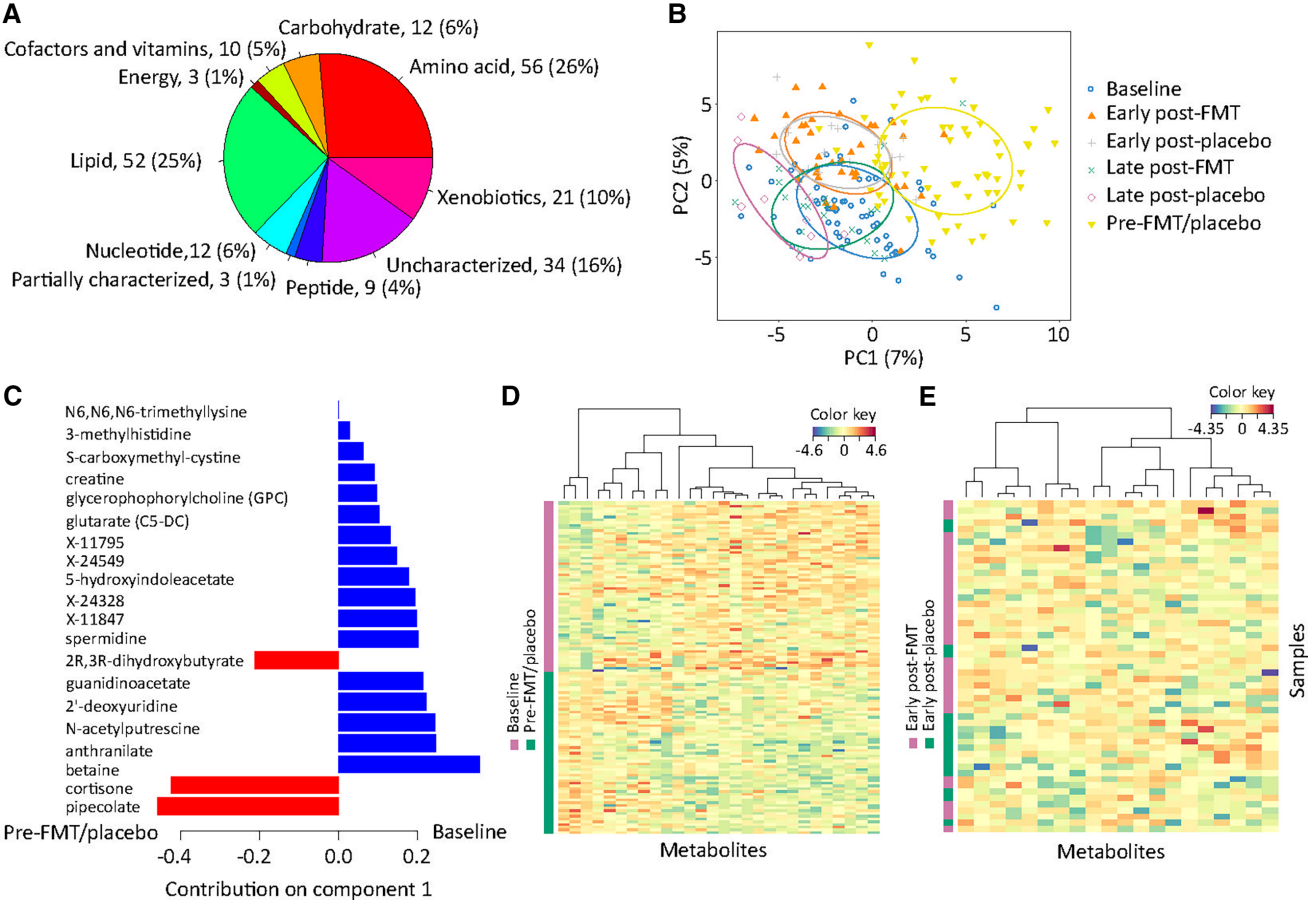
Serum metabolomics before and after FMT versus placebo. **A,** Distribution of serum metabolites across different pathways. **B,** PCA using logarithmically transformed metabolite levels, centered and scaled. 50% ellipses are shown. **C,** sPLS-DA loading plot for significant contributors to the first latent component in treatment arm-based classification. Each variable's contribution is shown by a vector. Vectors pointing to the right versus left represent differentially abundant metabolites in baseline versus pre-FMT/placebo samples, respectively. Longer vectors correspond to metabolites with more importance in group classification. **D,** Serum metabolomic heatmap among baseline and pre-FMT/placebo samples using the best classifying metabolites from sPLS-DA. Each row is a sample, and each column is a metabolite. The blue-red gradient shows metabolite levels scaled column-wise. **E,** Same analysis as in D, but for early post-FMT and early post-placebo samples. Logarithmically transformed metabolite values, Euclidean distance, and the complete method for hierarchical clustering were used to generate the heat maps in D and E. FMT: fecal microbiota transplantation, PC: principal component.

High-throughput profiling of the serum metabolome was performed using untargeted metabolomics of the blood samples. Of the 1,010 serum metabolites (202 samples), we excluded those with undetectable levels in >25% of the samples and kept only one member of every collinear metabolite set defined as having a Spearman correlation coefficient >0.5. Then, we imputed zeros in the remaining 212 metabolites by their half-minimum value across all samples. The distribution of these metabolites across different pathways is shown in Fig. 4A and their complete list is provided in Supplementary Table S1. Notably, 16% of the metabolites were uncharacterized and 1% were partially characterized molecules. The distribution of serum samples over time was baseline (*N* = 62), preintervention (*N* = 59), early postintervention (*N* = 53), and late postintervention (*N* = 28).

Principal component analysis (PCA) suggested a major change in the metabolome from baseline to preintervention (Fig. 4B), representing the period of most intense antibiotic exposures, nutritional changes, and intestinal toxicity of conditioning. The consequent loss of metabolically active microbiota (45, 46), dietary derived metabolites, and absorptive capacity of the intestines can explain the metabolomic shift. This was consistent with the results of sPLS-DA, where many metabolites were differentially abundant in baseline than pre-FMT/placebo samples (Fig. 4C), generating a perfect separation of the two groups (Fig. 4D). The two most differentially abundant metabolites (pipecolate and cortisone) were more abundant in pre-FMT/placebo samples. Pipecolate is a component or precursor of many microbial secondary metabolites but also a moiety of tacrolimus and sirolimus used as GVHD prophylaxis after transplantation (47), consistent with the sPLS-DA finding. Similarly, higher levels of the steroid hormone cortisone are expected to be present in the posttransplant period due to high stress-related conditions. Early post-FMT and early post-placebo samples were completely superimposed in metabolomic PCA (Fig. 4B and E), and sPLS-DA did not find differentially abundant metabolites. This finding argues against a major effect by FMT on the serum metabolome.

### A Novel Circulating Metabolite May Protect Against aGVHD

**FIGURE 5:**
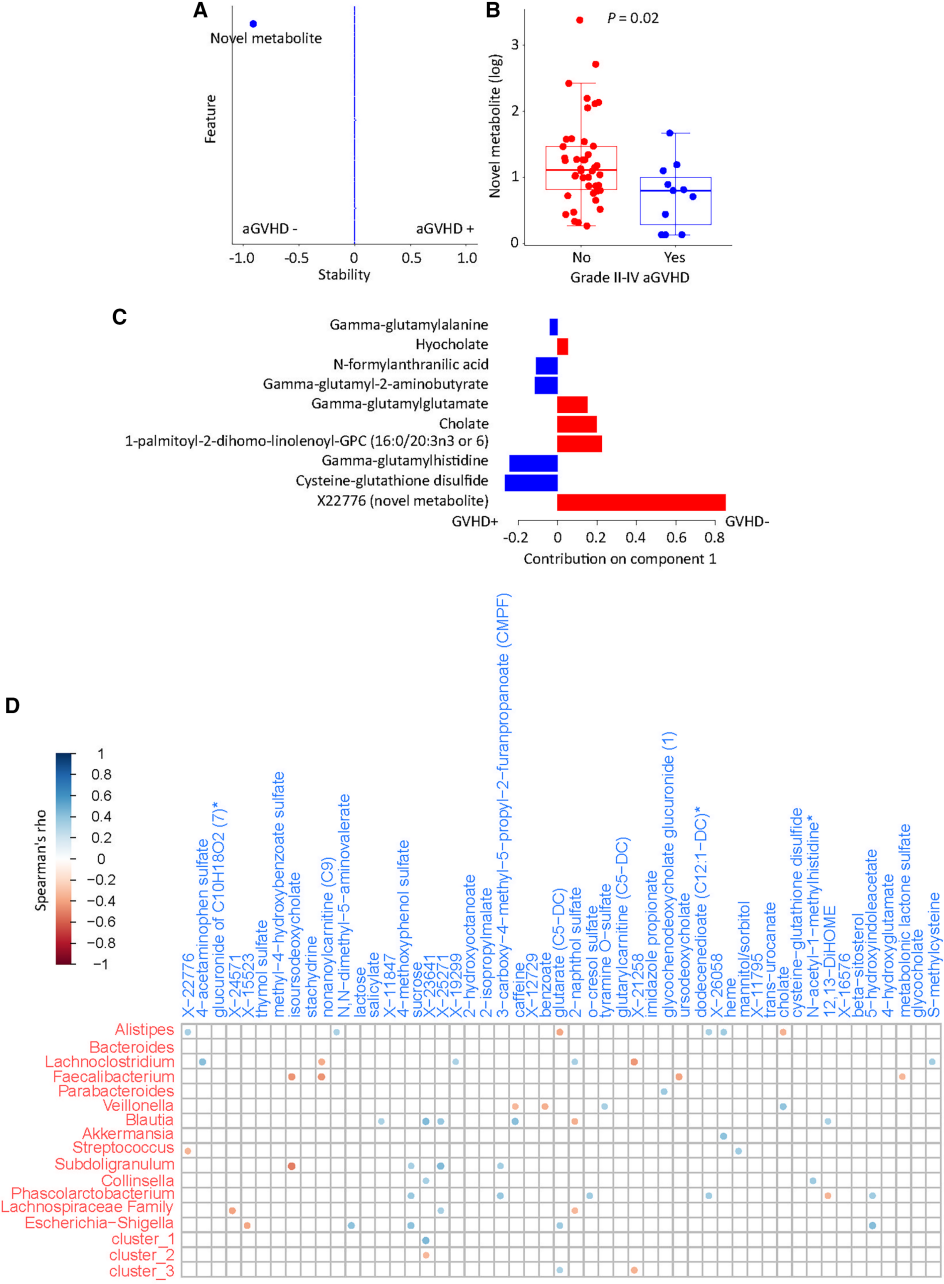
Early post-FMT/placebo serum metabolomics and subsequent aGVHD. **A,** Logistic LASSO regression to find early posttreatment serum metabolomic predictors of grade II–IV aGVHD. The *y* axis has all the features evaluated in the model including metabolites and the GVHD prophylactic regimen. Stability (*x* axis) indicates the proportion of the 100 cross-validated runs in which a given feature remained a significant predictor of GVHD. Positive/negative values show metabolites positively/negatively associated with aGVHD. Other than one metabolite negatively associated with aGVHD, all features were insignificant and formed an almost straight line with 0 stability. **B,** Association between the novel metabolite in LASSO and grade II–IV aGVHD. **C,** sPLS-DA loading plot for significant contributors to the first latent component in classification of aGVHD status. Each variable's contribution is shown by a vector. Vectors pointing to the right versus left represent differentially abundant metabolites in early post-FMT/placebo samples not preceding versus preceding grade II–IV aGVHD, respectively. Longer vectors correspond to metabolites with more importance in group classification. **D,** Multi-omics correlation plot. Spearman correlation coefficients (color gradient) are shown for statistically significant (*P* < 0.05) correlations. The top five taxa in each cluster (along with the clusters as individual units) and the first 50 metabolites with the highest variance among samples (along with the novel metabolite) were used in this analysis. Each box in B shows the median (horizontal middle line) and interquartile range. Whisker lines indicate nonoutlier maximum and minimum values. A small jitter is included for better visualization. *P* value is from a Wilcoxon test. aGVHD: acute graft-versus-host disease.

Although target organs in aGVHD are typically limited to the skin, gut, and liver, aGVHD is often associated with systemic manifestations such as fever, impaired hematopoiesis (48), and immunosuppression (49). Therefore, not only the gut microbiome but also the blood metabolome may be involved in aGVHD pathogenesis. Specifically, changes in the gut microbiome may alter the levels of specific blood metabolites, eliciting an immune attack against classic target organs, but also causing systemic manifestations of aGVHD. We thus asked whether the association between early post-FMT/placebo microbiota and aGVHD is partly mediated by blood metabolites. For a metabolite to be a mediator in the microbiota-aGVHD relationship, it needs to be associated with both aGVHD and microbiota. In logistic lasso to identify early post-FMT/placebo serum metabolites associated with subsequent grade II–IV aGVHD, only one metabolite was stably (and negatively) associated with aGVHD (Fig. 5A and B). This was an uncharacterized metabolite (a chlorine-containing molecule likely belonging to the xenobiotic pathway; personal communication with Metabolon, Inc.) as the only significant metabolite in 91 of the 100 runs of the algorithm. In sPLS-DA, the same metabolite emerged as the dominant predictor of no/grade I aGVHD (Fig. 5C). Together, this analysis identified a novel circulating metabolite with potential protective effects against aGVHD.

### Bile Acid Metabolism May be Involved in *Faecalibacterium*-aGVHD Association

Next, we performed cross-omics correlation analysis using both serum metabolites and microbial taxa to identify microbiota/metabolite associations in early post-FMT/placebo samples. The Spearman correlation coefficients and their corresponding *P* values for the top five taxa in each microbial cluster and the first 50 metabolites with the highest variance among samples were determined (Fig. 5D). Because of its unique association with grade II–IV aGVHD, our novel metabolite was added to the metabolites list. We paid special attention to *Faecalibacterium* as the genus with significant expansion after FMT and associated with grade II–IV aGVHD, asking whether it has relevant metabolomic correlates which could be mediating its GVHD association. *Faecalibacterium* abundance was significantly and negatively correlated with four metabolites, two of which were ursodeoxycholic acid (UDCA) and its epimer isoursodeoxycholic acid (Fig. 5D). UDCA is one of the main secondary bile acids produced by intestinal bacterial metabolism of host-derived primary bile acids. Several members of the gut microbiota were recently discovered to conjugate bile acids using amino acids (50). These bile acid conjugates (e.g., phenylalanocholic acid, tyrosocholic acid, and leucocholic acid) have agonistic effects on the farnesoid X receptor (FXR), can lead to reduced bile acid synthesis in the liver (50), and are enriched in patients with inflammatory bowel disease (50). As this line of research is very recent, the list of gut microbes capable of conjugating bile acids is likely to expand. *Faecalibacterium* may be one such bacteria, conjugating bile acids to metabolites that suppress bile acid production by the liver, subsequently leading to reduced secondary bile acids such as UDCA. Novel bile acid conjugates were not on our panel of characterized metabolites, thus we were not able to evaluate their association with *Faecalibacterium*. *Faecalibacterium* and the novel metabolite were not correlated.

## Discussion

Gut microbiota disruptions have been mechanistically linked with the risk of aGVHD. Examples include microbiota-mediated mucin degradation (14), modulation of MHC-II expression by intestinal epithelial cells (51), and M1 macrophage polarization induction by gut microbial choline metabolite (trimethylamine N-oxide; ref. 52). Although various indices of microbiota, considered to be indicators of health, were improved after FMT in our randomized trial, the incidence of grade II–IV aGVHD was numerically higher after FMT than placebo. This unexpected finding could be partly explained by the lack of balance between the two treatment arms in GVHD prophylaxis despite randomization. The small number of events prohibited a meaningful multivariable or subgroup analysis (16). The current work was an attempt to find a mechanistic explanation at the level of microbiota, the target of our randomized intervention. We performed extensive multi-omics analysis of longitudinal preintervention and postintervention gut microbiome and blood metabolome from samples collected in the trial. We evaluated changes in the blood metabolome associated with FMT and related changes in the gut microbiome. These analyses revealed several novel findings.

We identified microbial clusters within the gut microbiota that captured several genera previously proposed to influence the outcomes of alloHCT. These clusters were distinct in their physiology and host interactions. As an example, cluster 3 contains typical oral bacteria that are not an abundant part of the gut microbiota in healthy adults. Their expansion in the gut microbiota during alloHCT may reflect ectopic colonization via swallowed saliva, facilitated by a weak colonization resistance within the antibiotic-injured gut microbiome. Bacteria of oral origin such as *Streptococcus* commonly dominate the gut microbiota of alloHCT recipients (53) and are among the most frequently isolated genera from blood cultures of these patients. FMT diminished cluster 3, consistent with our previous analysis using an orthogonal approach (differential abundance analysis; ref. 16). Cluster 3 also included abundant members of the Proteobacteria phylum (e.g., *Escherichia-Shigella*) which are known causes of “Gram-negative sepsis” after transplantation, thus their clearance after FMT may decrease the risk of related bacteremia. Cluster 1, on the other hand, contained major members of the commensal gut microbiota, predominantly obligate anaerobic taxa. FMT led to the expansion of this cluster, again consistent with our previous differential abundance analysis (16). Cluster-based analysis indicated marked modulation of the gut microbiota after FMT. Interestingly, these cluster-level changes did not correlate well with grade II–IV aGVHD. Further analysis, using genus-level data, identified *Faecalibacterium* as a potential culprit for increased rates of aGVHD after FMT. As opposed to its parent cluster, this genus correlated both with the treatment arm and aGVHD. Specifically, *Faecalibacterium* was abundant among our healthy donors, less so among patients at baseline. The levels of this genus decreased during the early phase of transplantation, to later increase only after FMT. Importantly, early postintervention *Faecalibacterium* levels were strongly associated with subsequent grade II–IV aGVHD, suggesting a mediating role.

We did not find an effect by FMT on the serum metabolome. This contrasted with the period between baseline and pre-FMT/placebo timepoints when numerous significant changes in circulating metabolites were demonstrated. Impaired absorption of microbial metabolites due to chemotherapy- and radiation-induced intestinal toxicity may explain the absence of global changes in blood metabolome after FMT in our patient population. Significant changes in the blood metabolome after FMT may occur in other clinical settings with less severe or no intestinal damage such as metabolic syndrome (54). Nevertheless, we found a novel metabolite in blood which seemed protective against aGVHD. Precise characterization of this likely xenobiotic, its origin, and its mechanism of action could lead to new approaches for aGVHD prevention or treatment. The novel metabolite was not associated with the treatment arm, so it could not mediate the FMT-aGVHD link.

In multi-omics analysis, we found a negative correlation between *Faecalibacterium* and UDCA, a secondary bile acid with anti-inflammatory and cytoprotective effects (55). Whether and how *Faecalibacterium* may decrease UDCA levels in the blood cannot be determined from our data, but two hypotheses can be entertained. Amino acid conjugation of bile acid is a recently discovered metabolic function of the gut microbiota (50). The agonistic effect of these bile acids on FXR can decrease bile acid synthesis in the liver (50), leading to a smaller pool available for microbial metabolism to UDCA. The capacity of *Faecalibacterium* to conjugate bile acids is amenable to empiric testing. A decline in anti-inflammatory bile acids concurrent with *Faecalibacterium* expansion could potentially increase the risk of aGVHD. UDCA is commonly used to prevent hepatic complications including liver GVHD in patients with alloHCT (56, 57) and reduces colitogenic dysbiosis in mice (58). All patients in our HCT cohort received UDCA prophylaxis (same dose, schedule, and duration), eliminating a confounding effect related to the use of this medication. *Faecalibacterium* expansion was due to the engraftment of donor species and strains, consistent with our previous engraftment analysis demonstrating *Faecalibacterium* to have the second highest engraftment rate among all genera (16). Whether microbial neoantigens belonging to unique donor strains of *Faecalibacterium* elicited an alloimmune attack by the graft or a genus-level metabolic effect (e.g., via reductions in UDCA and other anti-inflammatory metabolites) led to increased aGVHD risk needs further research. *Faecalibacterium* is a potent butyrogenic commensal genus of the gut with a plethora of beneficial effects in homeostatic conditions (59). Our findings suggest that these effects may not be generalizable to the post-HCT setting.

One limitation of this study is lack of data on fecal metabolomics. As we did not perform shotgun metagenomic sequencing, we do not have a direct way to assess the metabolic potential of the microbiota and how it may impact the blood metabolome or clinical outcomes. This trial was designed when limited data were available on the connection between the gut microbiome and clinical outcomes after alloHCT. A systems biology approach in future studies using shotgun metagenomics combined with metatranscriptomics and fecal metabolomics would be informative. We used small-volume stool samples which although sufficient for sequencing, could not be used for bacterial culture. As a result, whether the presence of DNA represents concurrent presence of live bacteria cannot be ascertained. Our 16S rRNA amplicon method limited our taxonomic discovery to bacteria and only to the level of genus. Engraftment of specific *Faecalibacterium* strains could not be determined by this short-read method. As the role of nonbacterial components of the microbiome is becoming increasingly more apparent, shotgun or long-read techniques may provide novel insight into microbiota-mediated disease pathogenesis. In addition, specific subgroups could not be analyzed separately or adjusted for in multivariable analysis due to small sample size. Finally, although the highest level of causality and mechanistic evidence in human studies come from randomized controlled trials, the need for murine experiments under controlled environments and with few confounding variables is indispensable.

FMT is currently approved for the treatment of recurrent *Clostridium difficile* infection. This disease is a significantly less complex condition than the posttransplant setting and aGVHD. Severe intestinal macroenvironmental and microenvironmental injury, more extensive antibiotic exposure, nutritional changes, and the presence of an allogeneic immune system make alloHCT and aGVHD a unique setting. As FMT is a broad-spectrum, community-level intervention on the microbiota, its composition may need precision adjustment to match the complexity and unique aspects of alloHCT. A similar concept may also apply to the other conditions for which FMT is being tested. Our work supports context-specific design of the FMT product in future studies.

## Supplementary Material

Table S1 UPLC-MS/MS metabolites in serum
